# Real-world study on adverse drug reactions of pembrolizumab in endometrial cancer treatment: insights from the FAERS database

**DOI:** 10.3389/fphar.2025.1622339

**Published:** 2025-08-15

**Authors:** Huiping Zhang, Man Di, Jingjing Wang, Shan Wang, Yini Dai, Jingjing Huang, Zhuo Zhou

**Affiliations:** ^1^ Department of Obstetrics and Gynecology, Northwest University First Hospital, Xi’an, Shaanxi, China; ^2^ Department of Obstetrics and Gynecology, Tangdu Hospital, Air Force Medical University, Xi’an, Shaanxi, China; ^3^ Department of Pharmacy, Northwest University First Hospital, Xi’an, Shaanxi, China

**Keywords:** pembrolizumab, endometrial cancer, immunotherapy, immune checkpoint inhibitors, immune-related adverse events

## Abstract

**Objective:**

Pembrolizumab is a key drug in the immunotherapy of endometrial cancer (EC) and has improved the prognosis to some extent. However, adverse drug events (ADEs) have hindered the achievement of expected therapeutic outcomes in EC. This study, therefore, aims to investigate the ADEs of pembrolizumab using the FAERS database, offering new insights for clinical practice in EC treatment.

**Method:**

From the first quarter of 2016 to the first quarter of 2025, ADEs associated with pembrolizumab in EC were collected from the FAERS database. The Reporting Odds Ratio (ROR) was used as the primary analytical method for signal detection. To validate the robustness of the results, three additional algorithms—Proportional Reporting Ratio (PRR), Bayesian Confidence Propagation Neural Network (BCPNN), and Multi-Item Gamma Poisson Shrinker (MGPS)—were also applied. ADEs were systematically classified using the Medical Dictionary for Regulatory Activities (MedDRA) into System Organ Classes (SOC) and Preferred Terms (PT), and ranked by both frequency and signal strength.

**Results:**

A total of 2,154 ADEs associated with pembrolizumab in the treatment of EC were retrieved from the FAERS database. The age distribution of ADEs was primarily concentrated in the 65–85 years age group. The reported body weights were mainly in the 50–100 kg range. The most frequent ADE outcome was hospitalization. The majority of ADEs occurred within 0–30 days after pembrolizumab administration. Identified ADEs involved endocrine system disorders, including Increased Thyroid Hormones (ROR = 9.22), Decreased Thyroid Hormones (ROR = 5.31), and Immune-Mediated Hypothyroidism (ROR = 6.16). Skin and subcutaneous tissue disorders included Pruritic Rash (ROR = 3.16) and Blisters (ROR = 3.06). Liver-related issues included Increased Hepatic Enzymes (ROR = 2.25). These key signals were consistently confirmed by additional disproportionality algorithms, including PRR, BCPNN, and MGPS, reinforcing the robustness of the findings.

**Conclusion:**

This study used the FAERS database to identify frequently reported ADEs associated with pembrolizumab in the treatment of EC, including endocrine system diseases, musculoskeletal system disorders, skin and subcutaneous tissue reactions, and hepatotoxicity. These findings provide crucial evidence for risk stratification and safety monitoring in clinical practice, emphasizing the need for vigilance toward specific organ systems during the 0–30-day treatment window.

## 1 Introduction

EC is one of the most prevalent malignant tumors of the female reproductive system. Statistics show that in 2020, there were 417,000 new cases globally, and the incidence of EC has been rising steadily year by year (Crosbie et al., 2022). Despite ongoing advancements in treatment strategies for EC, the five-year survival rate for patients with advanced or recurrent EC remains around 20%–25%, contributing significantly to the global disease burden (van den Heerik et al., 2021; Tronconi et al., 2022). Chemotherapy remains the primary treatment for advanced or recurrent cases; however, due to molecular heterogeneity (such as POLE ultramutated, MSI-H, and TP53 mutated types), some patients develop resistance to treatment (Salmon et al., 2024). Furthermore, the tumor microenvironment contains a variety of immune-suppressive cells (e.g., regulatory T cells and myeloid-derived suppressor cells) and pro-tumor growth factors, which exacerbate resistance (Willvonseder et al., 2021). In recent years, immunotherapy has emerged as a promising treatment strategy, showing encouraging results in improving the prognosis of various malignant tumors (Cha et al., 2020). Immune checkpoint inhibitors (ICIs) are the most widely used form of immunotherapy, including PD-1, PD-L1, and CTLA-4 inhibitors (Szeto and Finley, 2019; Borgeaud et al., 2023). In the tumor microenvironment, EC exhibits higher PD-1/PD-L1 expression levels compared to other gynecological cancers, making the blockade of this pathway a potential therapeutic option for metastatic and recurrent diseases (Green et al., 2020). Pembrolizumab, a PD-1 inhibitor, is currently the most widely used ICIs in cervical cancer and has demonstrated significant efficacy, especially in patients with advanced or metastatic EC exhibiting high microsatellite instability (MSI-H) or mismatch repair deficiency (Harris, 2023). Multiple clinical trials have confirmed the substantial efficacy of pembrolizumab in patients with advanced or recurrent EC (Makker et al., 2023; Eskand et al., 2025). These results highlight the considerable potential of pembrolizumab in improving the prognosis of EC.

ICIs attack tumor cells by activating the immune system, but this treatment may also disrupt peripheral immune tolerance mechanisms, leading to abnormal activation of autoreactive T cells, which in turn attack normal tissues expressing specific antigens, triggering immune-related adverse events (irAEs) (Li et al., 2022). With the increased use of ICIs, the number of reported irAEs has surged in recent years, and these adverse events have, to some extent, affected the prognosis of cancer patients (Poto et al., 2022). Approximately 60% of irAEs cases are associated with three drugs: Ipilimumab, Nivolumab, and Pembrolizumab (Ramos-Casals et al., 2020). IrAEs can affect almost all tissues and organs, with the digestive system, endocrine system, and skin being the most frequently affected (Baxi et al., 2018). The adverse reactions caused by different types of immune checkpoint inhibitors also vary. CTLA-4 inhibitors often cause adverse reactions involving multiple organ systems and are generally more severe, especially in the gastrointestinal tract (Shepard et al., 2018). On the other hand, reactions caused by PD-1 inhibitors are usually more localized, with the skin, liver, and gastrointestinal tract being the most frequently affected areas (Suijkerbuijk et al., 2024). Currently, evidence on the efficacy of ICIs in the treatment of EC is primarily focused on therapeutic evaluation, while research on irAEs in this patient population remains significantly insufficient, and there is a lack of real-world-based adverse drug event reporting studies. Based on this, our study innovatively utilizes the FAERS database to systematically explore signals related to Pembrolizumab in the treatment of EC, particularly irAEs. These findings provide important data support for the development of personalized Pembrolizumab monitoring strategies in the clinical management of EC.

## 2 Materials and methods

### 2.1 Data collection

The data were sourced from the FAERS database, which can be downloaded from the official website of the U.S. FDA. In this study, the original ASCII data packages from the first quarter of 2016 to the first quarter of 2025. Were downloaded for data mining and statistical analysis. The original data download website is: https://fis.fda.gov/extensions/FPD-QDE-FAERS/FPD-QDE-FAERS.html.The main dataset comprised adverse drug event (ADE) reports from Q1 2016 to Q1 2025. To address the formal indication period, a sensitivity analysis restricted to the post-approval period (Q1 2021 onward) was also conducted, with results presented in the Supplementary Tables S1, S2.

### 2.2 Data deduplication

Since the data in this database is collected through spontaneous reporting, there may be duplicate reports or reports that have been withdrawn/deleted. This study strictly follows the FDA’s official guidelines for data cleaning. The data cleaning process is as follows: First, according to the deduplication method recommended by the FDA, we select the PRIMARYID, CASEID, and FDADT fields from the DEMO table and sort them in the order of CASEID, FDADT, and PRIMARYID. For reports with the same CASEID, the report with the largest FDADT value is retained. If both CASEID and FDADT are identical, the report with the largest PRIMARYID value is retained. Secondly, since the first quarter of 2019, each quarterly data package has included a list of deleted reports. After data deduplication, we remove the corresponding reports based on the CASEID from the deleted reports list. An example of the deduplication process is shown in Table 1. To ensure that the analysis focused exclusively on pembrolizumab used for the treatment of EC, we applied a strict restriction based on the reported therapeutic indication. Specifically, we filtered the INDI (indications) table in the FAERS database using the “INDI_PT” field and retained only those cases where the reported indication corresponded to one of the following MedDRA Preferred Terms: “Endometrial cancer”, “Endometrial carcinoma”, “Endometrioid adenocarcinoma”, “Uterine endometrioid adenocarcinoma”, “Uterine cancer” and “Endometrial neoplasm”. These PTs were selected based on clinical relevance and consistency with terminology used in EC diagnosis. All reports with missing or ambiguous indications (e.g., “cancer”, “malignant neoplasm”, or blank entries) were excluded from the final dataset to minimize misclassification bias. This restriction ensured that only cases with clearly specified EC-related indications were included for further disproportionality analysis.

**TABLE 1:** Example of deduplicated reports.

Primary id	Case id	FDA_DT	Operation
4271842	3070600	20060106	Delete
4271943	3070600	20060106	Delete
4283856	3070600	20060129	Delete
4314676	3070600	20060308	Retain

### 2.3 Application of the MedDRA dictionary

ADEs in the FAERS database were coded using the Medical Dictionary for Regulatory Activities (MedDRA version 27.1). Disproportionality analyses were primarily conducted at the Preferred Term (PT) level, with System Organ Class (SOC) level summaries used to group related PTs for improved clinical interpretability. To visually present the effect sizes and 95% confidence intervals of the top 30 signals at the PT level, forest plots were generated using the forestplot package in R, based on ROR estimates and supported by PRR, BCPNN, and MGPS algorithms.

### 2.4 Handling of false positive ADEs

The database includes a role cod field in the DRUG table to identify genuine “drug-adverse event” signals. The role cod is classified into four categories: PS (primary suspected drug), SS (secondary suspected drug), C (concomitant), and I (interaction). In this study, we identify cases in the DRUG file using the generic name (Pembrolizumab) of the drug and select the role cod as PS to improve accuracy. Additionally, to further limit the indications, we restrict the indication to endometrial cancer. To reduce the false positive rate, we apply the proportional imbalance measurement method to mine true “drug-adverse event” signals.

### 2.5 Statistical methods

This study uses R 4.4.2 for statistical analysis. To detect disproportionality signals, we employed the Reporting Odds Ratio (ROR) (Kerr et al., 2023; Papapetropoulos et al., 2024) as the primary analysis method. In addition, we applied three supplementary algorithms including Proportional Reporting Ratio (PRR) (Noguchi et al., 2020), Bayesian Confidence Propagation Neural Network (BCPNN) (Bate, 2007), and Multi-Item Gamma Poisson Shrinker (MGPS) (Ahlmann-Eltze and Huber, 2021) to validate and corroborate the signal robustness. Although the MHRA method was initially considered for inclusion in the disproportionality analysis, it was ultimately excluded due to its methodological redundancy with the PRR algorithm and the absence of added analytical value in our dataset. This decision was made to streamline the statistical approach and avoid overlap in signal detection criteria. These methods are statistical analysis techniques based on a 2 × 2 contingency table, which evaluate the statistical relationship between a specific drug and a specific ADE by calculating the relative frequency of the target adverse reaction in the database over a period of time. As shown in Table 2, a refers to the number of reports that contain both the target drug and the target drug’s adverse reaction; b refers to the number of reports containing the target drug but other drug-related adverse reactions; c refers to the number of reports with other drugs and the target drug’s adverse reactions; and d refers to the number of reports containing other drugs and other drug-related adverse reactions. The formulas and criteria for these five algorithms are presented in Table 3. This study was conducted in accordance with the REporting of A Disproportionality Analysis for DrUg Safety Signal Detection Using Individual Case Safety Reports in PharmacoVigilance (READUS-PV) guidelines (Fusaroli et al., 2024). The methodology strictly adhered to recommended standards for data preprocessing, disproportionality analysis, and signal strength interpretation. A completed READUS-PV checklist is provided in the Supplementary Material to ensure transparency and reproducibility.

**TABLE 2:** Proportional imbalance measurement method 2 × 2 table.

Types of drugs	Target adverse drug event reports	Other adverse drug event reports	Total
Target drug	a	b	a+b
Other drugs	c	d	c + d
Total	a+c	b + d	a+b + c + d

**TABLE 3:** Five main algorithms for evaluating the potential association between pembrolizumab and ADEs in EC.

Algorithms	Equation	Criteria
ROR	$95\%CI=e^{\ln\left(ROR\right)\pm \sqrt[{1.96}]{\left(\frac{1}{a}+\frac{1}{b}+\frac{1}{c}+\frac{1}{d}\right)}}ROR=\frac{ad}{bc}$	95% CI (lower limit) > 1, a ≥ 3
PRR	$PRR=\frac{a/\left(a+b\right)}{c/\left(c+d\right)}95\%CI=e^{\ln\left(PRR\right)\pm \sqrt[{1.96}]{\left(\frac{1}{a}-\frac{1}{a+b}+\frac{1}{c}-\frac{1}{c+d}\right)}}$	a ≥ 3, 95% CI (lower limit) > 1, and PRR ≥ 2
	χ2=ad−bc^2a+b+c+d/a+bc+da+cb+d	a≥3、PRR>2 and χ 2 ≥ 4
BCPNN	$IC=\log_{2}\frac{a\left(a+b+c+d\right)}{\left(a+c\right)\left(a+b\right)}$	IC-2SD > 0
MGPS	$\begin{array}{l} EBGM=a\left(a+b+c+d\right)/\left(a+c\right)/\left(a+b\right)\\ 95\%CI=e^{\ln\left(EBGM\right)\pm \sqrt[{1.96}]{\left(\frac{1}{a}+\frac{1}{b}+\frac{1}{c}+\frac{1}{d}\right)}} \end{array}$	EBGM05 > 2

### 2.6 Induction time of ADEs caused by pembrolizumab in EC

In these ADEs, some recorded the induction time. We used SPSS 29.0 to perform statistical analysis on the median and interquartile range of the induction time in these reports.

## 3 Results

### 3.1 Data acquisition results

**FIGURE 1:**
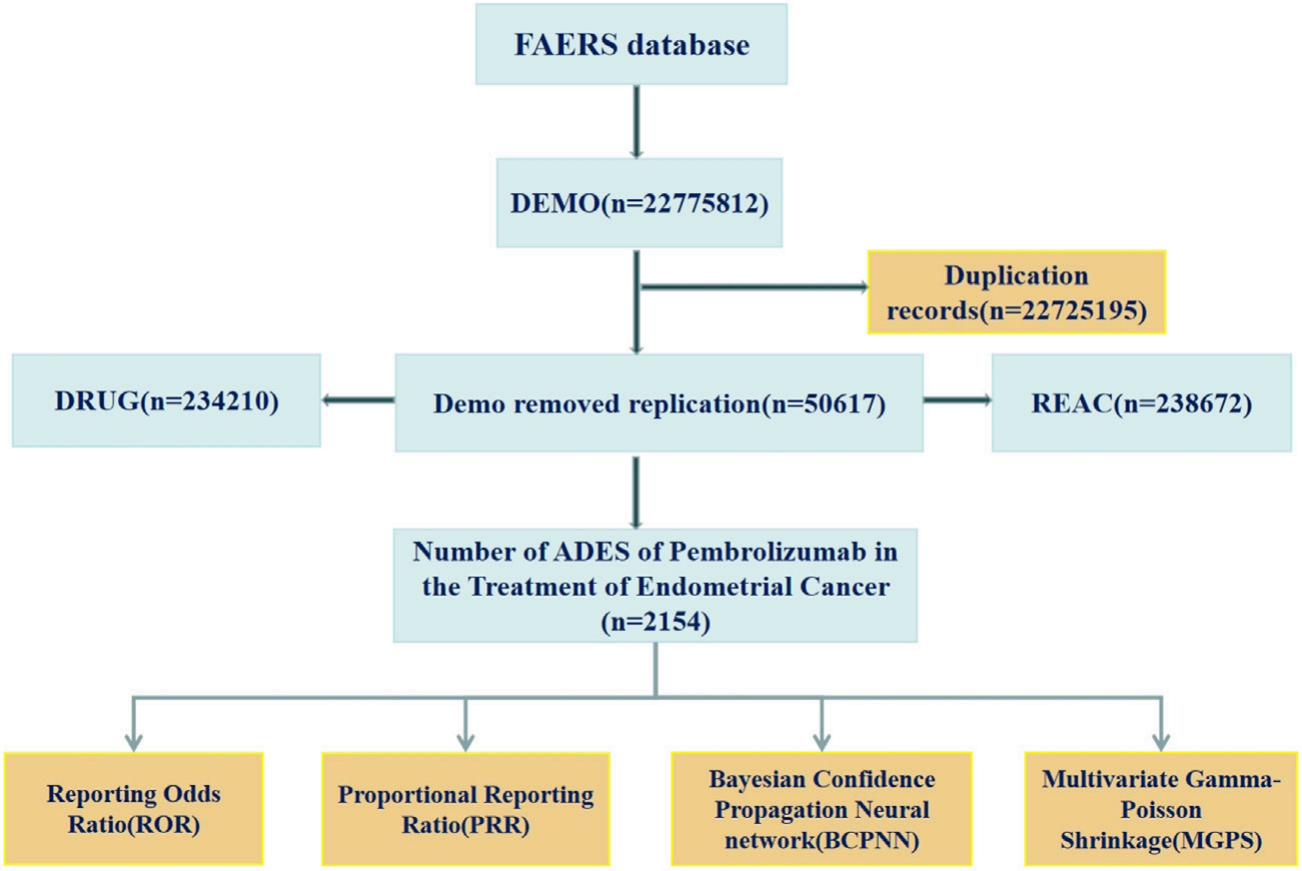
The flow diagram of screening reports containing pembrolizumab for the application in EC from the FAERS database.

From the first quarter of 2016 to the first quarter of 2025, a total of 2,154 ADEs related to the use of pembrolizumab in EC were retrieved from the FAERS database. These data include patients’ basic information (e.g., age, weight), names of ADEs, timing of ADEs, and outcomes of ADEs. The data acquisition process is illustrated in Figure 1.

### 3.2 Basic information on ADEs

**FIGURE 2:**
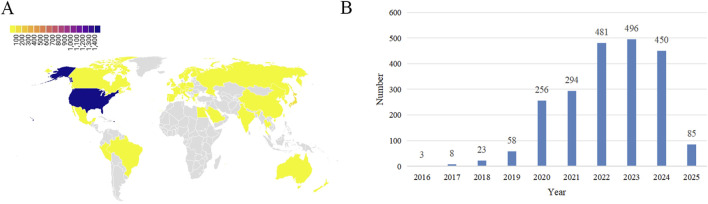
Basic information on ADEs related to the use of pembrolizumab in EC. **(A)** Distribution of ADEs by country; **(B)** Distribution of ADEs by year.

ADE reports for the use of pembrolizumab in EC come from 42 countries and regions. The country with the highest number of reports is the United States (75.72%), and the number of reports has shown an increasing trend over the years. The majority of reporters are patients and their families (56.04%), followed by doctors (27.53%) and healthcare professionals (12.86%). The age distribution of ADE reports is primarily concentrated between 65 and 85 years (30.22%), followed by 18–64.5 years (17.27%). The weight distribution of ADE reports is mainly between 50 and 100 kg (13.23%), followed by over 100 kg (2.60%) and under 50 kg (2.51%). The outcomes of reported ADEs are primarily hospitalization (18.48%) (as shown in Figure 2). Among these reports, serious cases account for approximately 52.83%, while non-serious cases account for approximately 47.17%. The method of administration is primarily intravenous (34.35%) (as shown in Table 4).

**TABLE 4:** General information on ADEs related to the use of pembrolizumab in EC.

Characteristics	Classification	N (%)
Weight (Kg)	<50 kg	54 (2.51%)
	50∼100 kg	285 (13.23%)
	>100 kg	56 (2.60%)
	Missing	1759 (81.66%)
Age (years)	<18 years	18 (0.84%)
	18∼64.9	372 (17.27%)
	65∼85	651 (30.22%)
	>85	6 (0.28%)
	Missing	1,107 (51.39%)
Reportertype	Consumer	1,207 (56.04%)
	Health Professional	277 (12.86%)
	Pharmacist	59 (2.74%)
	Physician	593 (27.53%)
	Missing	18 (0.84%)
Outcome	Death	163 (7.57%)
	Disability	23 (1.07%)
	Hospitalization	398 (18.48%)
	Life-Threatening	27 (1.25%)
	Other	1,543 (71.63%)
Serious and non-serious	Non-serious cases	1,016 (47.17%)
	Serious cases	1,138 (52.83%)
Death status	NO	1991 (92.43%)
	Yes	163 (7.57%)
Reported country	United States	1,631 (75.72%)
	Others	523 (24.28%)
Route of administration	Intravenous	740 (34.35%)
	Parenteral	1 (0.05%)
	Subcutaneous	1 (0.05%)
	Oral	9 (0.42%)
	Missing	1,403 (65.13%)

### 3.3 Frequency ranking results of ADEs

**FIGURE 3:**
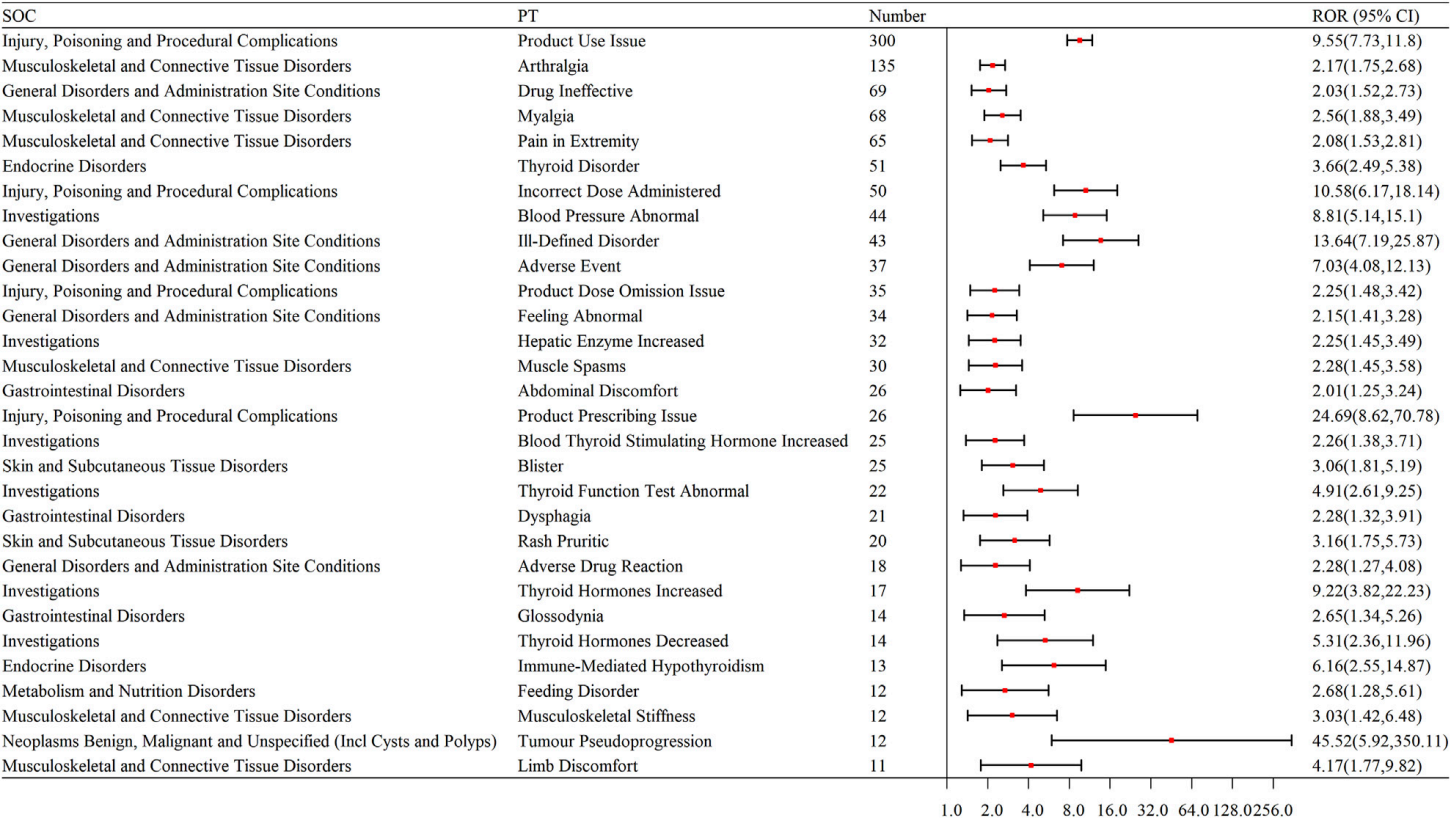
Forest plot of the top 30 signal frequencies of ADEs at the PT level for Pembrolizumab in the treatment of EC. SOC: System Organ Class; PT: Preferred Terms; ROR: Reporting Odds Ratio.

To enhance the representativeness of ADE signals, we selected the top 30 PTs based on their frequency and ROR values. Musculoskeletal and connective tissue disorders were particularly prominent, including Arthralgia (ROR = 2.17), Myalgia (ROR = 2.56), Pain in Extremity (ROR = 2.08), Musculoskeletal Stiffness (ROR = 3.03), and Limb Discomfort (ROR = 4.17). Endocrine system disorders also featured heavily, such as Thyroid Disorder (ROR = 3.66), Abnormal Thyroid Function Test (ROR = 4.91), Increased Thyroid Hormones (ROR = 9.22), and Immune-Mediated Hypothyroidism (ROR = 6.16). Other frequent signals included Pruritic Rash (ROR = 3.16), Blisters (ROR = 3.06), and Increased Hepatic Enzymes (ROR = 2.25) (Table 5; Figure 3).

**TABLE 5:** Top 30 signal frequencies of ADEs at the PT level for pembrolizumab in the treatment of EC.

PT	N	ROR (95%Cl)	PRR (X^2^)	EBGM (EBGM05)	IC (IC025)
Product Use Issue	300	9.55 (7.73, 11.8)	9.24 (647.79)	3.4 (2.85)	1.76 (1.55)
Arthralgia	135	2.17 (1.75, 2.68)	2.15 (53.38)	1.73 (1.45)	0.79 (0.5)
Drug Ineffective	69	2.03 (1.52, 2.73)	2.03 (23.47)	1.67 (1.31)	0.74 (0.34)
Myalgia	68	2.56 (1.88, 3.49)	2.55 (38.46)	1.93 (1.49)	0.95 (0.53)
Pain in Extremity	65	2.08 (1.53, 2.81)	2.07 (23.32)	1.69 (1.31)	0.76 (0.34)
Thyroid Disorder	51	3.66 (2.49, 5.38)	3.64 (50.01)	2.35 (1.7)	1.23 (0.74)
Incorrect Dose Administered	50	10.58 (6.17, 18.14)	10.52 (114.23)	3.52 (2.24)	1.82 (1.28)
Blood Pressure Abnormal	44	8.81 (5.14, 15.1)	8.77 (91.51)	3.34 (2.13)	1.74 (1.18)
Ill-Defined Disorder	43	13.64 (7.19, 25.87)	13.57 (109.41)	3.74 (2.19)	1.9 (1.32)
Adverse Event	37	7.03 (4.08, 12.13)	7.01 (66.95)	3.11 (1.97)	1.64 (1.03)
Product Dose Omission Issue	35	2.25 (1.48, 3.42)	2.25 (15.24)	1.78 (1.26)	0.83 (0.27)
Feeling Abnormal	34	2.15 (1.41, 3.28)	2.15 (13.32)	1.73 (1.22)	0.79 (0.22)
Hepatic Enzyme Increased	32	2.25 (1.45, 3.49)	2.24 (13.9)	1.78 (1.23)	0.83 (0.24)
Muscle Spasms	30	2.28 (1.45, 3.58)	2.27 (13.39)	1.8 (1.23)	0.84 (0.23)
Abdominal Discomfort	26	2.01 (1.25, 3.24)	2.01 (8.63)	1.66 (1.11)	0.73 (0.08)
Product Prescribing Issue	26	24.69 (8.62, 70.78)	24.62 (78.61)	4.15 (1.72)	2.05 (1.29)
Blood Thyroid Stimulating Hormone Increased	25	2.26 (1.38, 3.71)	2.25 (10.96)	1.79 (1.18)	0.84 (0.17)
Blister	25	3.06 (1.81, 5.19)	3.05 (19.15)	2.14 (1.37)	1.1 (0.41)
Thyroid Function Test Abnormal	22	4.91 (2.61, 9.25)	4.9 (29.81)	2.7 (1.59)	1.43 (0.68)
Dysphagia	21	2.28 (1.32, 3.91)	2.27 (9.37)	1.8 (1.14)	0.84 (0.12)
Rash Pruritic	20	3.16 (1.75, 5.73)	3.16 (16.09)	2.18 (1.32)	1.12 (0.36)
Adverse Drug Reaction	18	2.28 (1.27, 4.08)	2.27 (8.03)	1.8 (1.1)	0.84 (0.06)
Thyroid Hormones Increased	17	9.22 (3.82, 22.23)	9.2 (36.25)	3.39 (1.62)	1.76 (0.87)
Glossodynia	14	2.65 (1.34, 5.26)	2.65 (8.48)	1.97 (1.11)	0.98 (0.09)
Thyroid Hormones Decreased	14	5.31 (2.36, 11.96)	5.3 (20.38)	2.79 (1.42)	1.48 (0.54)
Immune-Mediated Hypothyroidism	13	6.16 (2.55, 14.87)	6.15 (21.39)	2.96 (1.42)	1.57 (0.58)
Feeding Disorder	12	2.68 (1.28, 5.61)	2.67 (7.37)	1.98 (1.07)	0.99 (0.03)
Musculoskeletal Stiffness	12	3.03 (1.42, 6.48)	3.03 (9.07)	2.13 (1.13)	1.09 (0.12)
Tumour Pseudoprogression	12	45.52 (5.92, 350.11)	45.45 (40.14)	4.42 (0.8)	2.14 (1.03)
Limb Discomfort	11	4.17 (1.77, 9.82)	4.17 (12.61)	2.51 (1.22)	1.33 (0.29)

PT: Preferred Terms; ROR: Reporting Odds Ratio; PRR: Proportional Reporting Ratio; IC025/IC-2SD: Information Component Lower Limit; EBGM05: Empirical Bayes Geometric Mean 5th Percentile.

To facilitate visual interpretation of effect sizes and their statistical precision, a forest plot (Figure 3) was constructed for the top 30 PTs using ROR values and 95% confidence intervals. These ROR-based signals were further validated using PRR, BCPNN, and MGPS algorithms. Most high-ranking PTs, such as Thyroid Disorder, Myalgia, and Immune-Mediated Hypothyroidism, also exceeded the detection thresholds for IC025 (>0) and EBGM05 (>2), demonstrating consistent signal direction and magnitude across algorithms and reinforcing the robustness of the findings.

To validate the robustness of our findings and address concerns regarding the inclusion of pre-approval data, we conducted a separate analysis restricted to the post-approval period for pembrolizumab in EC (from Q1 2021 to Q1 2025). The top 30 PT-level adverse event signals and baseline characteristics derived from this subset (Supplementary Tables S1, S2) revealed highly consistent results with the main analysis, both in signal ranking and strength across all four algorithms. These findings affirm that the overall signal trends are not artifacts of pre-approval data and remain stable in the real-world context after regulatory authorization.

### 3.4 Signal strength frequency ranking results of ADEs

**FIGURE 4:**
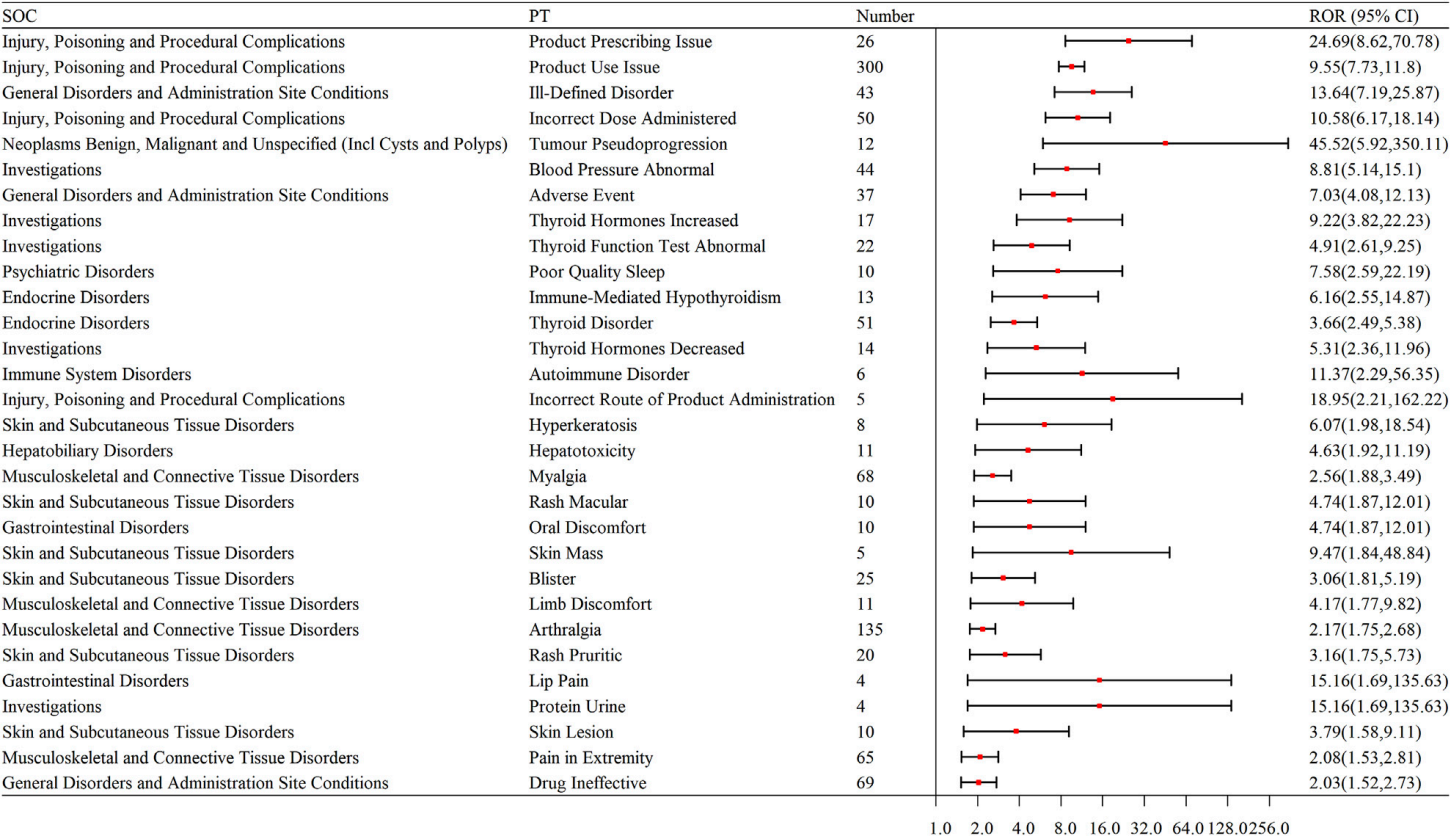
Forest plot of signal strength for the top 30 ADEs at the SOC level for pembrolizumab in the treatment of EC. SOC: System organ Class; PT: Preferred terms; ROR: Reporting odds ratio.

We selected the top 30 PTs based on signal strength, with the endocrine system being particularly prominent. Endocrine System: Immune-mediated hypothyroidism, thyroid disorder, increased thyroid hormones, decreased thyroid hormones, and abnormal thyroid function tests. Liver: Liver-related issues, hepatotoxicity. Skin and Skin-Related Issues: Rash, blisters, skin mass, pruritic rash, skin lesions, and hyperkeratosis. Musculoskeletal and Connective Tissue Disorders: Myalgia, arthralgia, pain in extremity, limb discomfort, and proteinuria. Other Adverse Events: Adverse events, drug ineffectiveness, oral discomfort, lip pain, and proteinuria (as shown in Table 6; Figure 4).

**TABLE 6:** Signal strength of the top 30 ADEs at the SOC level for pembrolizumab in the treatment of EC.

PT	N	ROR (95%Cl)	PRR (X^2^)	EBGM (EBGM05)	IC (IC025)
Product prescribing issue	26	24.69 (8.62, 70.78)	24.62 (78.61)	4.15 (1.72)	2.05 (1.29)
Product use issue	300	9.55 (7.73, 11.8)	9.24 (647.79)	3.4 (2.85)	1.76 (1.55)
Ill-defined disorder	43	13.64 (7.19, 25.87)	13.57 (109.41)	3.74 (2.19)	1.9 (1.32)
Incorrect dose administered	50	10.58 (6.17, 18.14)	10.52 (114.23)	3.52 (2.24)	1.82 (1.28)
Tumour pseudoprogression	12	45.52 (5.92, 350.11)	45.45 (40.14)	4.42 (0.8)	2.14 (1.03)
Blood pressure abnormal	44	8.81 (5.14, 15.1)	8.77 (91.51)	3.34 (2.13)	1.74 (1.18)
Adverse event	37	7.03 (4.08, 12.13)	7.01 (66.95)	3.11 (1.97)	1.64 (1.03)
Thyroid hormones increased	17	9.22 (3.82, 22.23)	9.2 (36.25)	3.39 (1.62)	1.76 (0.87)
Thyroid function test abnormal	22	4.91 (2.61, 9.25)	4.9 (29.81)	2.7 (1.59)	1.43 (0.68)
Poor quality sleep	10	7.58 (2.59, 22.19)	7.58 (19.03)	3.19 (1.3)	1.67 (0.54)
Immune-mediated hypothyroidism	13	6.16 (2.55, 14.87)	6.15 (21.39)	2.96 (1.42)	1.57 (0.58)
Thyroid disorder	51	3.66 (2.49, 5.38)	3.64 (50.01)	2.35 (1.7)	1.23 (0.74)
Thyroid hormones decreased	14	5.31 (2.36, 11.96)	5.3 (20.38)	2.79 (1.42)	1.48 (0.54)
Autoimmune disorder	6	11.37 (2.29, 56.35)	11.36 (14.18)	3.59 (0.94)	1.84 (0.39)
Incorrect route of product administration	5	18.95 (2.21, 162.22)	18.94 (14.16)	3.99 (0.66)	2 (0.39)
Hyperkeratosis	8	6.07 (1.98, 18.54)	6.06 (13)	2.95 (1.16)	1.56 (0.33)
Hepatotoxicity	11	4.63 (1.92, 11.19)	4.63 (14.09)	2.63 (1.26)	1.4 (0.35)
Myalgia	68	2.56 (1.88, 3.49)	2.55 (38.46)	1.93 (1.49)	0.95 (0.53)
Rash macular	10	4.74 (1.87, 12.01)	4.73 (13.1)	2.66 (1.22)	1.41 (0.32)
Oral discomfort	10	4.74 (1.87, 12.01)	4.73 (13.1)	2.66 (1.22)	1.41 (0.32)
Skin mass	5	9.47 (1.84, 48.84)	9.47 (10.82)	3.42 (0.87)	1.77 (0.22)
Blister	25	3.06 (1.81, 5.19)	3.05 (19.15)	2.14 (1.37)	1.1 (0.41)
Limb discomfort	11	4.17 (1.77, 9.82)	4.17 (12.61)	2.51 (1.22)	1.33 (0.29)
Arthralgia	135	2.17 (1.75, 2.68)	2.15 (53.38)	1.73 (1.45)	0.79 (0.5)
Rash pruritic	20	3.16 (1.75, 5.73)	3.16 (16.09)	2.18 (1.32)	1.12 (0.36)
Lip pain	4	15.16 (1.69, 135.63)	15.15 (10.57)	3.83 (0.61)	1.94 (0.19)
Protein urine	4	15.16 (1.69, 135.63)	15.15 (10.57)	3.83 (0.61)	1.94 (0.19)
Skin lesion	10	3.79 (1.58, 9.11)	3.79 (10.26)	2.39 (1.15)	1.26 (0.19)
Pain in extremity	65	2.08 (1.53, 2.81)	2.07 (23.32)	1.69 (1.31)	0.76 (0.34)
Drug ineffective	69	2.03 (1.52, 2.73)	2.03 (23.47)	1.67 (1.31)	0.74 (0.34)

PT: preferred terms; ROR: reporting odds ratio; PRR: proportional reporting ratio; IC025/IC-2SD: information component lower limit; EBGM05: Empirical Bayes Geometric Mean 5th Percentile.

### 3.5 Induction time of ADEs caused by pembrolizumab in EC

**FIGURE 5:**
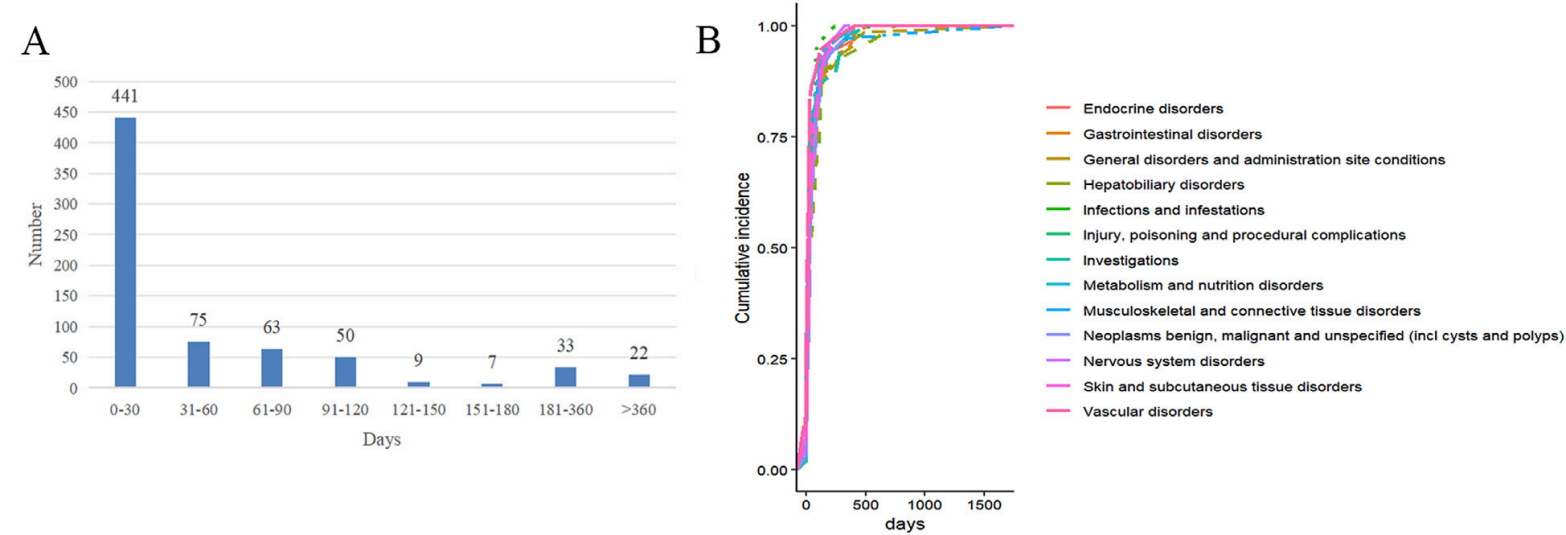
The occurrence time of ADEs of pembrolizumab in EC. **(A)** Time interval distribution of adverse reaction onset; **(B)** Cumulative risk timeline of adverse reactions at the SOC level.

Among the ADEs reports, approximately 700 cases include the onset times of adverse reactions, with the highest number of reports (441 cases) occurring within 0–30 days. This suggests a high probability of occurrence during the early stages of drug treatment (as shown in Figure 5). The median onset time for endocrine system adverse reactions is 20 days, for gastrointestinal disorders it is 21 days, for general disorders and administration site conditions it is 20 days, for hepatobiliary disorders it is 34 days, and for musculoskeletal and connective tissue disorders it is 25 days (as shown in Figure 5; Table 7).

**TABLE 7:** Induction time distribution of ADEs caused by pembrolizumab in EC at the SOC level.

SOC	Numer	Median (day)	Q1 (day)	Q3 (day)
Endocrine disorders	32	20.00	7.00	59.50
Gastrointestinal disorders	82	21.00	7.00	83.25
General disorders and administration site conditions	68	20.00	7.00	47.25
Hepatobiliary disorders	29	34.00	11.5	115.00
Injury, poisoning and procedural complications	60	25.50	14.25	48.75
Investigations	46	20.00	6.50	39.50
Metabolism and nutrition disorders	31	26.00	7.00	73.00
Musculoskeletal and connective tissue disorders	33	25.00	8.50	49.50
Neoplasms benign, malignant and unspecified (incl cysts and polyps)	31	32.00	13.00	80.00
Nervous system disorders	52	31.00	8.25	78.00
Skin and subcutaneous tissue disorders	47	25.00	11.00	56.00
Vascular disorders	38	12.00	3.00	27,50

SOC: system organ class; Median: the value in the middle of the data set; Q1: 25th Percentile; Q3: 75th Percentile.

## 4 Discussion

ICIs have become a major approach in the treatment of malignant tumors, but their efficacy is somewhat limited due to the presence of immune-related toxicities (Durbin et al., 2025). Pembrolizumab, a highly selective PD-1 inhibitor, has been widely used in this field since its FDA approval for MSI-H/dMMR advanced EC in 2021 (Maio et al., 2022). However, its application in EC is relatively recent, and the adverse reactions associated with its use in EC are still unclear. Therefore, this study systematically investigated and analyzed the frequently adverse reaction signals associated with pembrolizumab in EC using the FAERS database, providing valuable insights for evaluating the clinical safety of pembrolizumab in the treatment of EC.

ICIs work by relieving the tumor’s suppression of the immune system, enhancing the body’s immune system’s ability to recognize and attack the tumor, particularly in cases where traditional therapies have limited efficacy or resistance. They have shown significant therapeutic advantages (Lentz et al., 2021). However, the associated irAEs remain an important concern. In this study, pembrolizumab was notably associated with endocrine system-related adverse reactions in EC, followed by skin tissue lesions, musculoskeletal disorders, hepatotoxicity, nephritis, and others. These ADEs suggest that pembrolizumab’s application in EC may trigger widespread autoimmune responses affecting multiple organ systems. Therefore, elucidating its potential mechanisms, strengthening early detection, and optimizing management strategies are critical to improving its clinical safety.

Endocrine diseases induced by ICIs are the most frequently irAEs. Approximately 30%–40% of patients treated with ICIs experience thyroid dysfunction, while type 1 diabetes and adrenal insufficiency are relatively rare (Wright et al., 2021). The incidence of thyroid dysfunction is about 10% in patients treated with PD-1/PD-L1 inhibitors alone, 5% in those treated with CTLA-4 inhibitors alone, and 15%–20% in those receiving ICIs combination therapy (Keam et al., 2024). A prospective study on ICIs treatment showed that the average onset times for thyroiditis and hypothyroidism were 42 ± 19 days and 103 ± 78 days, respectively, suggesting that thyroiditis may occur earlier than hypothyroidism (Iwama et al., 2022). However, the exact mechanisms behind thyroid dysfunction following ICIs treatment remain unclear. In a mouse model, administration of anti-PD1 antibodies combined with thyroid globulin induced thyroid-related immune ADEs, with CD4^+^ T cells expressing granzyme B directly damaging thyroid follicular cells (Yasuda et al., 2021). Another study revealed that in patients who developed thyroid disease after ICIs treatment, histopathological analysis showed that T cell activation led to damage of thyroid follicular structures, accompanied by significant CD8^+^ T cell infiltration, indicating that T cell-mediated cytotoxicity is primarily driven by cytotoxic CD8^+^ T cells (Zaborowski et al., 2020). Furthermore, Th1 cytokines (such as IFN-γ and TNF-α) induce apoptosis of thyroid follicular cells, triggering transient thyroiditis with elevated thyroid peroxidase antibodies (TPOAb), which may eventually progress to permanent hypothyroidism. This suggests that monitoring serum thyroid hormone levels and dynamically tracking thyroid autoantibodies (TPOAb, TgAb) could improve risk stratification (Wu et al., 2023). In our study, we also observed the occurrence of immune-mediated hypothyroidism (ROR = 6.16), increased thyroid hormones (ROR = 9.22), and decreased thyroid hormones (ROR = 5.31) in patients with EC treated with pembrolizumab. Additionally, the median onset time for endocrine diseases in our study was 20 days, providing valuable insights for personalized treatment.

Previous studies have shown that the incidence of ICIs related dermatologic ADEs is approximately 30%–60%, with clinical manifestations including morbilliform or lichenoid rashes, vitiligo, pruritus, herpes-like diseases, and psoriasis-like or eczematous dermatitis (Quach et al., 2021). A retrospective study revealed that around 17% of patients treated with PD-1 inhibitors developed lichenoid reactions and eczema (Hwang et al., 2016). One hypothesis suggests that there may be shared antigens between tumor cells and normal tissues, such as the skin, which can be recognized by antigen-specific T cells. This enhances T cell recognition of skin antigens, leading to immune-related skin ADEs (Berner et al., 2019). Skin biopsies from patients receiving anti-PD-1 treatment showed infiltration and activation of CD8^+^ T cells at the dermal-epidermal junction, releasing cytotoxic factors that damage keratinocytes (Goldinger et al., 2016). The release of cytokines is closely associated with skin-related immune ADEs. In another study on melanoma, patients who developed rashes after ICI treatment had significantly higher baseline serum levels of angiopoietin-1 (Ang-1) and CD40L compared to those without rashes. Additionally, patients who developed dermatitis showed decreased levels of plasma CX3CL1, vascular endothelial growth factor-alpha (VEGF-α), and MHCI-related peptides 1–3 months after ICI treatment (Tyan et al., 2021). Another retrospective study showed that increased levels of eosinophils, serum IL-6, IL-10, and immunoglobulin E in peripheral blood were closely associated with the exacerbation of skin toxicity (Phillips et al., 2019), suggesting that these cytokines may be potential therapeutic targets for skin-related adverse reactions. In our study, we also observed dermatologic ADEs, primarily including hyperkeratosis (ROR = 6.07), blisters (ROR = 3.06), and rashes (ROR = 4.74). The median time for the appearance of skin-related ADEs was 25 days, providing a time reference for clinical management. Currently, corticosteroids can alleviate symptoms of ICIs related skin ADEs to some extent, but in serious cases, it may be necessary to discontinue ICIs treatment or use immunosuppressive therapy (Thompson et al., 2021).

In this study, we observed muscle-related ADEs in patients treated with pembrolizumab for EC, including limb discomfort (ROR = 4.17), myalgia (ROR = 2.56), and other musculoskeletal ADEs. The median time to onset of musculoskeletal ADEs was 25 days, providing valuable insights for clinical management. A retrospective study found that approximately 12.7% of cancer patients treated with ICIs experienced musculoskeletal irAEs, with joint pain and myalgia being the most frequent (9.8%) (Melia et al., 2023). Research has shown that muscle biopsy characteristics in patients with immune-related myositis include the presence of T cells and macrophages, with minimal B cell infiltration (Pinal-Fernandez et al., 2023). This suggests that the immune system activated by PD-1 inhibitors may attack normal musculoskeletal tissue, leading to an inflammatory response in the musculoskeletal system (Fletcher and Johnson, 2024). Additionally, muscle pain and joint pain may also result from the drug’s effect on the neural network, causing abnormal sensation and altered pain signal transmission (Marini et al., 2021). For mild cases, non-steroidal anti-inflammatory drugs (NSAIDs) and corticosteroids may be used to alleviate symptoms. In more serious cases, discontinuation of immune therapy and immunosuppressive treatment may be considered (Postow et al., 2018). Immune-mediated hepatotoxicity (IMH) refers to liver damage caused by the abnormal activation of the immune system during ICIs treatment, with an incidence of about 2%–25% (Xing et al., 2023). Clinical manifestations of IMH include mild elevation of liver enzymes, autoimmune hepatitis, cholangitis, jaundice, and liver failure (Remash et al., 2021). IMH is related to the dose and type of ICIs, with higher doses increasing the risk of hepatotoxicity (Jennings et al., 2019). Furthermore, a meta-analysis showed that PD-1 inhibitors are more likely to cause hepatotoxicity compared to PD-L1 inhibitors (Fu et al., 2021). IMH results from excessive immune activation in liver tissue, and steroids are the first-line treatment. If necessary, discontinuing ICIs treatment or administering immunosuppressive therapy may help alleviate symptoms (Dara and De Martin, 2025). In our study, the onset of pembrolizumab-related hepatotoxicity occurred at a median time of 34 days, further providing useful reference for clinical management.

Currently, pembrolizumab is the most widely used ICIs in EC, and several clinical trials have shown that it improves the prognosis of EC to some extent (O'Rourke, 2022). In previous studies, combination therapy with ICIs had a higher incidence of irAEs compared to monotherapy, and the onset of these events occurred earlier (Hammond et al., 2022). The incidence of irAEs is also related to the type of ICIs used. Generally, PD-1 and PD-L1 inhibitors are better toA. Time interval distribution of adverse reaction onset; B. Cumulative risk timeline of adverse reactions at the SOC level. lerated than CTLA-4 inhibitors (Blum et al., 2023). This study provides an in-depth discussion of the ADEs associated with pembrolizumab in the treatment of EC, offering a theoretical basis for the clinical management of immunotherapy in EC.

However, this study has some limitations. The data from the FAERS database are based on spontaneous reports, leading to a significantly higher reporting rate for serious or novel adverse events compared to mild or frequently events, which may distort risk signals. In addition, reports in the FAERS database usually lack detailed clinical information, such as disease severity, comorbidities, and medication history, making comprehensive drug safety assessment more complex.

## 5 Conclusion

Pembrolizumab is the most widely used drug in the immunotherapy of EC, but research on its ADEs in large real-world samples is still relatively scarce. This study conducted a systematic analysis of pembrolizumab’s ADEs in EC based on the FAERS database and identified frequently ADEs signals associated with pembrolizumab. The study highlights the detection window for ADEs occurring within the first 30 days, and further discusses the timing of ADEs across different systems, providing important reference information for clinical drug use. These ADEs involve the endocrine system, musculoskeletal system, skin and subcutaneous tissue, hepatotoxicity, and others, offering valuable insights for clinical safety. However, this study also has certain limitations. Although disproportionality analysis has some value in assessing the strength of signals, it cannot quantify the risk or prove causality between adverse reactions and the targeted drug, which presents challenges in determining the relationship between the two. In the future, further research based on multi-center, large-sample clinical data to explore the mechanisms of pembrolizumab’s ADEs in EC and develop corresponding diagnostic and treatment plans will help improve its safety evaluation and provide stronger support for clinical practice.

## Data Availability

The original contributions presented in the study are included in the article/Supplementary Material, further inquiries can be directed to the corresponding author.
