# Supplementary material for: Real-world study on adverse drug reactions of pembrolizumab in endometrial cancer treatment: insights from the FAERS database
Source: Front Pharmacol. 2025 Aug 15;16:1622339. doi: 10.3389/fphar.2025.1622339 (PMC12394486; doi:10.3389/fphar.2025.1622339)
Supplement: Supplementary file 1 [file Table1.docx]

**Table S1 General Information on AEs Related to the Use of Pembrolizumab in EC (Q1 2021–Q1 2025).**

| **Characteristics** | **classification** | **N (%)** |
| --- | --- | --- |
| Weight (Kg) | ＜50kg | 22 (1.4%) |
|  | 50～100kg | 192 (12.4%) |
|  | ＞100kg | 47 (3.0%) |
|  | Missing | 1291 (83.2%) |
| Age(years) | <18years | 1 (0.1%) |
|  | 18～64.9 | 241 (15.5%) |
|  | 65～85 | 473 (30.5%) |
|  | >85 | 4 (0.3%) |
|  | missing | 833 (53.7%) |
| Reportertype | Consumer | 912 (58.8%) |
|  | Health Professional | 197 (12.7%) |
|  | Pharmacist | 404 (26.0%) |
|  | Physician | 37 (2.4%) |
|  | Missing | 2 (0.1%) |
| Outcome | Death | 61 (3.9%) |
|  | Disability | 11 (0.7%) |
|  | Hospitalization | 237 (15.3%) |
|  | Life-Threatening | 5 (0.3%) |
|  | Other | 432 (27.8%) |
|  | missing | 806 (51.9%) |
| Serious and non-serious | Non-serious cases | 806 (51.9%) |
|  | Serious cases | 746 (48.1%) |
| Death status | NO | 1491 (96.1%) |
|  | Yes | 61 (3.9%) |
| reported country | United States | 1230 (79.3%) |
|  | Others | 322(21.7%) |

**Table S2 Top 30 Signal Frequencies of AEs at the PT Level for Pembrolizumab in the Treatment of EC (Q1 2021–Q1 2025).**

| PT | N | ROR (95%Cl) | PRR(X2) | EBGM(EBGM05) | IC(IC025) |
| --- | --- | --- | --- | --- | --- |
| product use issue | 268 | 32.06 ( 21.7 - 47.38 ) | 30.61 ( 735.1 ) | 3.8 ( 2.57 ) | 1.93 ( 1.66 ) |
| arthralgia | 104 | 2.17 ( 1.69 - 2.78 ) | 2.15 ( 38.48 ) | 1.69 ( 1.31 ) | 0.75 ( 0.4 ) |
| drug ineffective | 55 | 2.6 ( 1.82 - 3.72 ) | 2.59 ( 29.74 ) | 1.88 ( 1.31 ) | 0.91 ( 0.41 ) |
| pain in extremity | 50 | 2.4 ( 1.66 - 3.46 ) | 2.39 ( 23.17 ) | 1.79 ( 1.24 ) | 0.84 ( 0.32 ) |
| incorrect dose administered | 46 | 12.35 ( 6.54 - 23.33 ) | 12.26 ( 98.64 ) | 3.33 ( 1.76 ) | 1.74 ( 1.08 ) |
| thyroid disorder | 45 | 3.37 ( 2.21 - 5.12 ) | 3.35 ( 36.31 ) | 2.15 ( 1.41 ) | 1.1 ( 0.53 ) |
| myalgia | 44 | 2.72 ( 1.82 - 4.07 ) | 2.71 ( 25.73 ) | 1.92 ( 1.29 ) | 0.94 ( 0.38 ) |
| inappropriate schedule of product administration | 44 | 3.45 ( 2.25 - 5.29 ) | 3.43 ( 36.7 ) | 2.17 ( 1.42 ) | 1.12 ( 0.54 ) |
| blood pressure abnormal | 33 | 9.64 ( 4.87 - 19.09 ) | 9.59 ( 63.62 ) | 3.15 ( 1.59 ) | 1.65 ( 0.88 ) |
| ill-defined disorder | 33 | 15.16 ( 6.7 - 34.28 ) | 15.07 ( 76.02 ) | 3.46 ( 1.53 ) | 1.79 ( 0.99 ) |
| hepatic enzyme increased | 28 | 2.36 ( 1.45 - 3.85 ) | 2.36 ( 12.62 ) | 1.78 ( 1.09 ) | 0.83 ( 0.13 ) |
| product prescribing issue | 25 | 20.07 ( 6.98 - 57.69 ) | 19.99 ( 62.27 ) | 3.62 ( 1.26 ) | 1.86 ( 0.9 ) |
| thyroid function test abnormal | 21 | 6.12 ( 2.95 - 12.71 ) | 6.1 ( 30.85 ) | 2.75 ( 1.33 ) | 1.46 ( 0.52 ) |
| blister | 19 | 2.77 ( 1.5 - 5.12 ) | 2.76 ( 11.48 ) | 1.95 ( 1.05 ) | 0.96 ( 0.08 ) |
| adverse event | 18 | 19.24 ( 5.67 - 65.35 ) | 19.19 ( 44.37 ) | 3.6 ( 1.06 ) | 1.85 ( 0.7 ) |
| adverse drug reaction | 16 | 2.56 ( 1.33 - 4.95 ) | 2.56 ( 8.45 ) | 1.87 ( 0.97 ) | 0.9 ( -0.05 ) |
| rash pruritic | 14 | 3.2 ( 1.53 - 6.72 ) | 3.2 ( 10.59 ) | 2.1 ( 1 ) | 1.07 ( 0.01 ) |
| bone pain | 14 | 2.24 ( 1.13 - 4.44 ) | 2.24 ( 5.65 ) | 1.73 ( 0.87 ) | 0.79 ( -0.21 ) |
| dyspepsia | 14 | 2.13 ( 1.08 - 4.2 ) | 2.13 ( 5.05 ) | 1.68 ( 0.85 ) | 0.75 ( -0.24 ) |
| thyroid hormones increased | 13 | 8.33 ( 2.97 - 23.38 ) | 8.31 ( 23.25 ) | 3.03 ( 1.08 ) | 1.6 ( 0.32 ) |
| thyroid hormones decreased | 12 | 4.8 ( 1.96 - 11.76 ) | 4.8 ( 14.44 ) | 2.52 ( 1.03 ) | 1.33 ( 0.1 ) |
| visual impairment | 12 | 2.4 ( 1.14 - 5.08 ) | 2.4 ( 5.6 ) | 1.8 ( 0.85 ) | 0.85 ( -0.25 ) |
| joint swelling | 11 | 2.52 ( 1.14 - 5.54 ) | 2.51 ( 5.61 ) | 1.85 ( 0.84 ) | 0.89 ( -0.27 ) |
| tumour pseudoprogression | 11 | 35.24 ( 4.55 - 273.03 ) | 35.17 ( 30.45 ) | 3.85 ( 0.5 ) | 1.94 ( 0.4 ) |
| glossodynia | 10 | 2.46 ( 1.08 - 5.62 ) | 2.46 ( 4.9 ) | 1.83 ( 0.8 ) | 0.87 ( -0.34 ) |
| lymphadenopathy | 9 | 3.6 ( 1.39 - 9.34 ) | 3.6 ( 7.95 ) | 2.22 ( 0.86 ) | 1.15 ( -0.22 ) |
| skin lesion | 9 | 5.76 ( 1.93 - 17.2 ) | 5.76 ( 12.64 ) | 2.7 ( 0.9 ) | 1.43 ( -0.05 ) |
| musculoskeletal stiffness | 9 | 2.62 ( 1.08 - 6.32 ) | 2.62 ( 4.95 ) | 1.89 ( 0.78 ) | 0.92 ( -0.37 ) |
| liver function test increased | 9 | 2.4 ( 1.01 - 5.7 ) | 2.4 ( 4.2 ) | 1.8 ( 0.76 ) | 0.85 ( -0.42 ) |
| hepatotoxicity | 9 | 3.2 ( 1.27 - 8.07 ) | 3.2 ( 6.8 ) | 2.1 ( 0.83 ) | 1.07 ( -0.27 ) |
